# Worry and fear as predictors of fatalism by COVID-19 in the daily work of nurses

**DOI:** 10.1590/1518-8345.5833.3545

**Published:** 2022-08-01

**Authors:** Jhon Alex Zeladita-Huaman, Roberto Zegarra-Chapoñan, Rosa Castro-Murillo, Teresa Catalina Surca-Rojas

**Affiliations:** 1 Universidad Nacional Mayor de San Marcos, Departamento Académico de Enfermería, Lima, Peru.; 2 Universidad María Auxiliadora, Escuela Profesional de Enfermería, Lima, Peru.; 3 Ministerio de Salud, Escuela Nacional de Salud Pública, Lima, Peru.

**Keywords:** Fear, Coronavirus Infections, Nurses, Mental Health, Fatal Outcome, Activities of Daily Living, Medo, Infecções por Coronavirus, Enfermeiras e Enfermeiros, Saúde Mental, Evolução Fatal, Atividades Cotidianas, Miedo, Infecciones por Coronavirus, Enfermeras y Enfermeros, Salud Mental, Resultado Fatal, Actividades Cotidianas

## Abstract

**Objective:**

to analyze the relationship between the concern and fear of COVID-19 with fatalism in the daily work of nurses.

**Method:**

analytical cross-sectional study carried out with a total of 449 nurses. Data collection was performed using instruments validated in Peru. In the analysis, the Shapiro-Wilk test and the Spearman correlation coefficient were used, and two multiple regression models were estimated, with variable selection in stages.

**Results:**

nurses had a moderate level of fatalism and a low level of fear and concern about COVID-19. The first statistical model, which included sociodemographic variables, explains only 3% of the fatalism variance. However, a second model that includes fear and perception explains 33% of it.

**Conclusion:**

Worry, fear and having been diagnosed with COVID-19 were predictors of fatalism. It is suggested the implementation of psycho-emotional interventions in daily work - aimed at Nursing professionals who present high levels of fear or concern - to reduce fatalism and prevent fatal consequences of the pandemic and promote health.

## Resumo

**Objetivo:**

analisar a relação entre a preocupação e o medo da COVID-19 com o fatalismo no cotidiano de trabalho dos enfermeiros.

**Método:**

estudo transversal analítico, realizado com 449 enfermeiros. A coleta de dados foi realizada por meio de instrumentos validados no Peru. Na análise, foram utilizados o teste de Shapiro-Wilk e o coeficiente de correlação de Spearman, sendo estimados dois modelos de regressão múltipla, com seleção de variáveis por etapas.

**Resultados:**

os enfermeiros apresentaram nível moderado de fatalismo e baixo nível de medo e preocupação com a COVID-19. O primeiro modelo estatístico, que incluiu variáveis sociodemográficas, explica apenas 3% da variância de fatalismo. No entanto, um segundo modelo que inclui medo e percepção explica 33%.

**Conclusão:**

a preocupação, o medo e ter sido diagnosticado com COVID-19 foram fatores preditores de fatalismo. Sugere-se a implementação de intervenções psicoemocionais no cotidiano de trabalho, voltadas para profissionais de Enfermagem que apresentem altos níveis de medo ou preocupação, para reduzir o fatalismo e, assim, prevenir consequências fatais da pandemia e promover a saúde.

## Resumen

**Objetivo:**

analizar la relación entre la preocupación y el miedo al COVID-19 con el fatalismo, en lo cotidiano laboral de los enfermeros.

**Método:**

estudio transversal tipo analítico, realizado en 449 enfermeros. La recolección de datos se realizó mediante instrumentos validados en Perú. En el análisis se utilizó la prueba de Shapiro-Wilk, el coeficiente de correlación de Spearman y se estimaron dos modelos de regresión múltiple, con selección de variables por pasos.

**Resultados:**

los enfermeros presentaron un nivel moderado de fatalismo y un nivel bajo de miedo y preocupación por la COVID-19. El primer modelo estadístico que incluyó las variables sociodemográficas apenas explica un 3% de la varianza de fatalismo; sin embargo, un segundo modelo que incluye el miedo y la percepción explica el 33%.

**Conclusión:**

la preocupación, el miedo y el haber sido diagnosticado con COVID-19 fueron factores predictores de fatalismo. Se sugiere implementar intervenciones psicoemocionales en el cotidiano laboral - focalizadas en profesionales de Enfermería que presenten altos niveles de miedo o preocupación - para reducir el fatalismo y de esta manera prevenir consecuencias fatales de la pandemia y promover la salud.

**Highlights**

(1) Worry and fear are predictors of COVID-19 fatalism.

(2) Nurses have a moderate level of fatalism by COVID-19.

(3) Nurses have a low level of fear and concern about COVID-19.

(4) Having had COVID-19 is a predictor of fatalism by COVID-19.

(5) The importance of mental preparation of health teams.

## Introduction

The COVID-19 pandemic showed that, in addition to a simple health crisis, there were signs of a real civilizational or “social” crisis in everyday life^1^, which not only affected the physical and mental health of the population, but also disrupted essential mental health services in 93% of the countries in the world^2^. In this context, compared to other health workers, nurses are at greater risk of developing trauma or psychiatric disorders^3^.

The impact of COVID-19 on nurses’ mental health is related to the daily conditions of their work and the fear of contracting the disease and infecting their family members^4^; this consequence could trigger a state of psychological and physical tension capable of activating pathological behaviors^5^, which would increase the risk of committing suicide^6^. In fact, recent evidence from a retrospective study suggests that psychological distress and extreme fear of COVID-19 were stressors in cases of suicide during quarantine^7^.

One of the psychological constructs little investigated during the pandemic is fatalism by COVID-19, conceptualized as the perception or belief that a person has about possible situations after being infected by this virus^8^. Thus, a study in Peru indicated that this perception is associated with sex, age, religion and risk of infection^9^. In addition, among people living in the United States, it was reported that fatalistic messages increased the fatalism score, while optimistic messages reduced it^10^.

Fear of COVID-19 is defined as an unpleasant emotional state, triggered by the perception of threatening stimuli^11^. Recent studies have shown differences in the type of association that exists between this emotional state and fatalism. Fear of COVID-19 was inversely associated with fatalism (destiny would be determined externally)^12^ and with the fight against coronavirus^10^. However, a study that evaluated its relationship with three fatalism subscales reported that two of them - predetermination and luck - affect negatively, in contrast to pessimism, which impacts positively^13^. On the other hand, no studies were found that evaluated the association between fatalism and concern about COVID-19, conceptualized as an emotional response to this disease and which constitutes an important aspect for its management^14^.

Research on fatalism in nurses’ daily work is relevant, as this perception not only increases psycho-emotional stress, but also represents a barrier to the adoption of preventive and health-promoting measures. Thus, a study indicates that fatalism predicts the refusal to adopt preventive behaviors in the face of COVID-19^15^. Likewise, predetermination - one of the dimensions of fatalism - is associated with participation in activities with a risk of contracting this disease^13^. 

It is known that the fatal consequences of COVID-19 in nurses can be prevented through interventions focused on their risk factors^16^. However, there are few studies that analyze the factors associated with fatalism. The following research question was raised: what is the relationship between the concern and fear of COVID-19 with fatalism, considering the possibility of being infected with this virus, in the daily work of nurses? Thus, this study aims to analyze the relationship between the concern and fear of COVID-19 with fatalism in the daily work of nurses.

## Method

### Study design

Analytical cross-sectional quantitative study, guided by the Standards for Quality Improvement Reporting Excellence (SQUIRE) tool.

### Setting

The study was carried out in the city of Lima, Peru. Nurses who work in public (hospitals, health centers) and private (clinics, outpatient clinics) health facilities were invited to participate.

### Population and sample

The sample consisted of 449 nurses who reported performing some care work activity at the time of the research. They were selected through intentional sampling. Nurses who did not live in Peru and those who did not have access to electronic devices connected to the Internet were excluded.

### Instrument

The research technique was used through a virtual questionnaire. In the first section of the instrument, the informed consent form was requested, which included a question to consult the desire to participate in the study. In the second section, questions were asked about the sociodemographic characteristics considered as possible predictive factors: age, sex, vaccination status, length of professional experience, workplace, remote work and whether the patient was diagnosed with COVID-19.

To measure fatalism, the scale of fatalism at the possibility of being infected with coronavirus (F-COVID-19 Scale) was used^8^. This scale measures the perception/belief of possible situations after the contagion of this disease. The scale was validated to be applied to a Peruvian population in 2020 and showed adequate reliability (Cronbach’s alpha of 0.70). Their answer alternatives ranged from 1 = strongly disagree to 5 = strongly agree, so that the higher the score on the scale, the greater the fatalistic perception. This scale is composed of two subscales: extreme fatal consequences as a result of infection (4 questions) and concern about the consequences of coronavirus infection (3 questions). The scale allows obtaining a total score and a score for each factor of the subscale^8^. Furthermore, in this investigation, adequate reliability was found (McDonald’s Omega coefficient of 0.77). This coefficient was preferred over Cronbach’s Alpha, because McDonald’s Omega coefficient is a more robust measure against violation of assumptions in instrument measurement^17^.

Fear of COVID-19 was assessed using the Fear of COVID-19 Scale (FCV-19S/FCV-19S Scale)^11^. This scale, built on an extensive literature review, was validated in a Peruvian population in 2020 and showed an optimal level of internal consistency (Comparative Fit Index of 0.988). It has a total of 7 questions and its alternatives range from 1 = strongly disagree to 5 = strongly agree. Therefore, the higher the score on the scale, the higher the fear score. This scale is composed of two subscales: emotional reactions of fear (7 questions) and somatic expressions of fear (3 questions). In addition, it allows obtaining a total score and a score for each subscale^11^. Thus, in this study, it was found to have adequate reliability (McDonald’s Omega coefficient of 0.87). 

Concern about the contagion of the COVID-19 was assessed using a Likert PRE-COVID-19 Scale^14^, which was adapted from a cancer concern scale and validated in a Peruvian population in 2020; consists of six questions whose answer alternatives range from 1 = never or rarely to 4 = almost always, so that the higher the scale score, the greater the concern^14^. As with the other scales, in this study it was found to have adequate reliability (McDonald’s Omega coefficient of 0.85). It is important to emphasize that, to be used in this study, it had the approval of its authors. However, as the scales were used in nurses, they were previously submitted to the approval of specialist professionals who worked in Nursing specializing in mental health and health in general. These were members of rapid response teams to COVID-19 in different areas of Peru.

### Data collection procedures

Data collection was carried out using a form created on Google Forms, which was disseminated on the social networks of health institutions, in scientific nursing societies and in universities that offer postgraduate degrees in Nursing programs, between April and June 2021.

### Data statistical analysis 

At first, to meet the objectives of this study, the descriptive statistical results of the variables studied were analyzed. In this analysis, the assumption of normality of continuous variables was revised using the Shapiro-Wilk test, identifying that none of the variables followed a normal distribution. Subsequently, to estimate the bivariate correlations, the Spearman correlation coefficient was used. The interpretation of the effect was made considering that: correlations close to 0.10 are small, those close to 0.30 are moderate and those close to 0.50 are strong^18^.

Finally, two regression models were estimated, with selection of variables in stages^19^, to identify the model with the best capacity to predict the fatalism score. The first stage included control variables such as age, sex, vaccination status, whether they work remotely and whether they were diagnosed with COVID-19. The second stage included, in addition to the control variables, concern and fear. Both models were compared in terms of R^2^ (Coefficient of Determination) and the model was interpreted with a significantly higher level of explained variance. For both models, the assumptions of normality of the residuals were revised using the comparative graphical method with a half-normal distribution^20^. This method allows to graphically diagnose the adjustment of the residuals of the models against a theoretical normal distribution. Furthermore, the assumption of homogeneity of the variances of both models was verified with the Breusch-Pagan test, which when presenting statistically significant statistics, indicated the presence of heteroscedastic residues^21^. Likewise, the multicollinearity of both models was revised using the Variation Inflation Factor (VIF). According to this indicator, variables that obtain scores greater than 10 are considered problematic^22^. The existence of outliers was explored by calculating Cook’s D indicator; when this indicator has scores higher than 1, it is considered a case with high influence and high residuals, for which it is considered a probable outlier. The software R v 4.1.0^23^ was used in all analyses.

### Ethical aspects

The study was approved by the Research Ethics Committee of the *Universidad María Auxiliadora* (draft number 002-2021). The recommendations were respected and the ethical principles indicated in the Declaration of Helsinki established by the Peruvian regulations were complied with.

## Results

In total, 456 nursing professionals responded, seven participants were excluded because they were from other countries. The remaining 449 were eligible, with a mean age of 37.9 years old (SD = 11.1). According to the State/Region in which they lived, 75.5% (339) lived in Lima, 8.0% (36) in Ayacucho, 2.9% (13) in Callao, 2.9% (13) in Junín and 10.7% (48) in 15 other cities in Peru.

As for the characteristics of the Nursing professionals interviewed, 88.4% were women and 11.6% were men. Regarding vaccination against COVID-19, 19.2% did not receive any vaccine, 6.2% received the first dose and 74.6% completed the second dose. As for professional experience, 37.4% had less than 5 years, 26.9% between 5 and 10 years, 12.3% between 11 and 15 years and 23.4% over 15 years. Likewise, only 18.7% did remote work and 40.3% were diagnosed with COVID-19 at some point in the pandemic. According to the type of work center, 70.6% worked in a hospital or clinic, 19.2% in a first level health care facility and 10.2% in an establishment that offers ambulance service or home care.

Table 1 shows the average score of the variables studied and the diagnosis of normality based on the Shapiro-Wilk test. The coronavirus fatalism score obtained (21.35 SD 4.07) would indicate that nurses have a moderate level in this variable (score close to the intermediate value of the scale). Regarding the score of concern (12.12 SD 3.52) and fear of COVID-19 (16.55 SD 5.42), both would indicate a low level in these variables under study (score lower than the intermediate value of the scale). On the other hand, it can be observed that in all cases the variables present a significant *p*-value for the normality test, indicating that the null hypothesis of normal distribution is rejected for all study variables (*p*<0.01).

**Table 1:** Results of descriptive statistics and the Shapiro-Wilk normality test for the dimensions fatalism, concern about COVID-19 and dimensions of fear of nursing professionals (n=449). Lima, LIM, Peru, 2021

	Mean (SD*)	SW^†^	P value
Fatalism subscale: concern about the consequences of contagion	11.7 (2.18)	0.92	<0.001
Fatalism subscale: extreme fatal consequences	9.65 (2.73)	0.98	<0.001
Fatalism total scale	21.35 (4.07)	0.99	0.04
Scale of concern for COVID-19	12.12 (3.52)	0.96	<0.001
Fear subscale: emotional reactions to fear	11.24 (3.72)	0.98	<0.001
Fear subscale: Somatic expressions of fear	5.31 (2.30)	0.87	<0.001
Total scale of fear	16.55 (5.42)	0.98	<0.001

*SD = Standard Derivation; ^†^SW = Shapiro-Wilk Normality Test

Bivariate analysis (Table 2) reported that all matrix correlations are positive and significant. Analyzing the relationships of each of the dimensions of fatalism with the other variables in the study, it was possible to observe that the subscale of fatalism (concern about the consequences of contagion) has a strong correlation with the scale of concern about COVID-19 (Rho = 0.41). In addition, it has a moderate correlation with the fear subscale (emotional fear reactions) (Rho = 0.34), a mild correlation with the somatic expressions of the fear subscale (Rho = 0.14) and a moderate correlation with total fear (Rho = 0.29). Likewise, the fatalism (extreme fatal consequences) subscale has a strong correlation with the COVID-19 concern scales (Rho = 0.49), emotional fear reactions (Rho = 0.50) and the total fear scale (Rho = 0.50), while it has a moderate correlation with the somatic expressions of the fear scale (Rho = 0.37). Finally, the total fatalism scale shows strong correlations with the scales: concern about COVID-19 (Rho = 0.52), emotional fear reactions (Rho = 0.50) and total fear scale (Rho = 0.48), while it has a moderate correlation with the somatic expressions of the fear scale (Rho = 0.32).

**Table 2:** Spearman’s correlations between the dimensions fatalism, concern and fear of Nursing professionals (n=449). Lima, LIM, Peru, 2021

Scales and subscales	SEF1*	SEF2^†^	EF^‡^	EP4^§^	SEM1^||^	SEM2^¶^
SEF1*	1	-	-	-	-	-
SEF2^†^	0.41**	1	-	-	-	-
EF^‡^	0.77**	0.88**	1	-	-	-
EP^§^	0.41**	0.49**	0.52**	1	-	-
SEM1^||^	0.34**	0.50**	0.50**	0.66**	1	-
SEM2^¶^	0.14**	0.37**	0.32**	0.47**	0.59**	1
Total scale of fear	0.29**	0.50**	0.48**	0.66**	0.95**	0.81**

*SEF1 = Fatalism subscale: concern about the consequences of contagion; ^†^SEF2 = Fatalism Subscale: Extreme Fatal Consequences; ^‡^EF = Total Fatalism Scale; ^§^EP = COVID-19 Concern Scale; ^||^SEM1 = Fear subscale: emotional reactions of fear; ^¶^SEM2 = Fear subscale: somatic expressions of fear; ^**^P value < 0.01

Table 3 shows the regression models estimated to predict the full scale of fatalism. It is observed that the R^2^ of model 1 is equal to 0.03, which indicates that the sociodemographic variables explain only 3% of the total variance of fatalism. In contrast, model 2 has an R^2^ equal to 0.36, indicating that, when the worry and total fear scales are included, they explain 33% of the fatalism variance. This increase in variance is statistically significant (*p* < 0.001), so only model 2 is interpreted, in which it is observed that respondents who were diagnosed with COVID-19 had a score greater than 0.72 points in the average of fatalism in relation to those not diagnosed with the virus, this is a mild effect (*B* = 0.09, *p* < 0.05). Also, for each point increase on the concern scale, 0.46 points are added to the total fatalism scale, this is a moderate effect (*B* = 0.39, *p* < 0.001). Finally, for each point increase on the fear scale, fatalism increases by 0.17 points, this is a mild to moderate effect (*B* = 0.23, *p* < 0.001).

**Table 3:** Non-standardized regression coefficients, standard errors and standardized coefficients of stepwise regression models for predicting the total scale of fatalism in nursing professionals (n=449). Lima, LIM, Peru, 2021

	Model 1				Model 2			
	b*	*EE* ^†^	*B* ^‡^	*p* ^§^	b*	*EE* ^†^	*B* ^‡^	*p* ^§^
Cut-off	20.84	0.97		<0.001	12.21	0.98		<0.001
Age	0.01	0.02	0.04	0.46	0.02	0.01	0.06	0.15
Sex (Female)	0.29	0.60	0.02	0.63	0.25	0.49	0.02	0.61
Vaccination Status (1^st^ dose)	0.59	0.88	0.04	0.50	-0.21	0.72	-0.01	0.78
Vaccination Status (2^nd^ dose)	-0.69	0.50	-0.07	0.16	-0.62	0.41	-0.07	0.13
Remote Work (Yes)	-0.85	0.51	-0.08	0.09	-0.56	0.41	-0.05	0.17
COVID-19 diagnosis (Yes)	0.98	0.39	0.12	0.01	0.72	0.32	0.09	0.02
Concern					0.46	0.06	0.39	<0.001
Fear: Total					0.17	0.04	0.23	<0.001
R^2^	0.03			0.03	0.36			<0.001
Delta R^2^					0.33			<0.001

*b = Non-standardized coefficient; ^†^SE = Standard Error; ^‡^B = Standard coefficient; ^§^ *p* = Statistical significance

**Figure 1:**
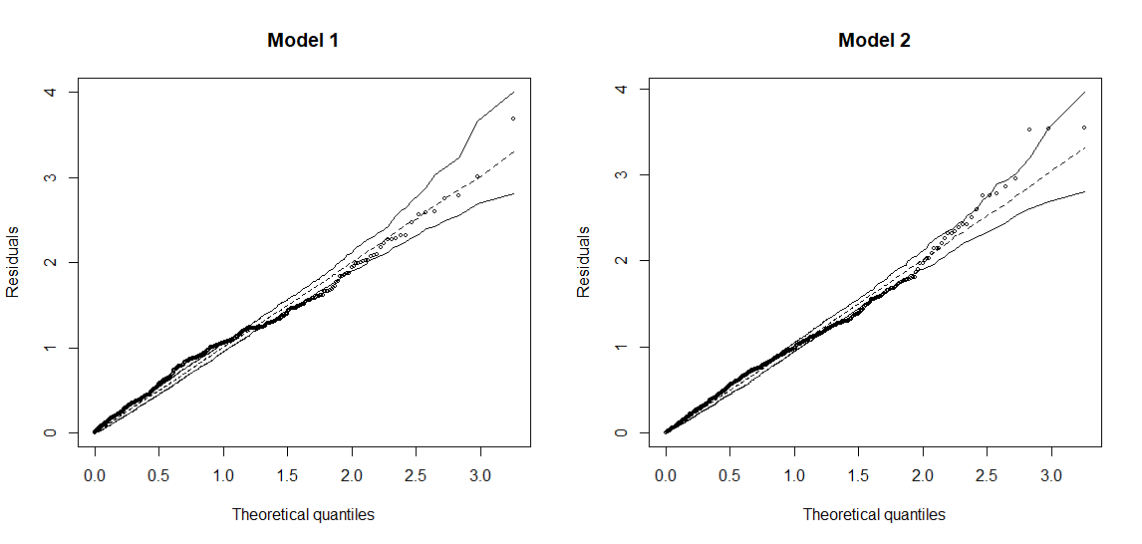
Diagnostic graphic of residual normality assumption with the comparative graphical method with half-normal distribution for regression models for predicting fatalism in Nursing professionals (n=449). Lima, LIM, Peru, 2021

Figure 1 shows the diagnosis of the residual normality assumption for the fatalism regression models. It is observed that, in both models, all cases of adjusted residuals fall within the simulation bands of a half-normal distribution. This indicates that, for both models, the residuals fit a normal univariate distribution.

Regarding the assumption of homogeneity of variances, the Breusch-Pagan test for Model 1 was equal to 5.56, with 6 degrees of freedom and a *p*-value of 0.48. This value is not statistically significant, indicating that the model variance is homogeneously distributed along the entire regression line. Likewise, the Breusch-Pagan test for Model 2 was equal to 9.65, with 8 degrees of freedom and a *p*-value of 0.29, which is also not statistically significant. Thus, in this model it is also concluded that the variance is homogeneous along the regression line.

Finally, with regard to the multicollinearity assumption, it was found that all variables in both models have scores between 1 and 2 points, which indicates that the predictor variables of both models do not have strong correlations that could affect the accuracy of the estimation of its parameters.

Finally, in relation to the diagnosis of atypical cases, all cases included in the analysis had scores below 0.04, indicating that no case in the collected sample has sufficient influence or residues to be considered problematic cases or outliers*.*

## Discussion

This study analyzed the fatalism of Nursing professionals living in 19 of the 24 departments/provinces of Peru, it was carried out during the first months of the beginning of vaccination, a period in which the number of cases and deaths by COVID-19 remained high, both in general population and health professionals. As a main result, it is reported that worry, fear and having been diagnosed with COVID-19 were predictors of fatalism in a model that explains 33% of the variability. We know that this is possibly the first study that analyzed the predictors of fatalism in nurses’ daily work. This finding is important and significant to understand the psychological, emotional and behavioral reactions of this group of professionals, in times of the COVID-19 pandemic, and illuminates the actions to be taken to improve the psychosocial condition of the group.

One of the findings of this study refers to the fact that the increase in the fear score of COVID-19 was associated with an increase in the score on the fatalism scale when faced with the possibility of becoming infected with the coronavirus. This finding is consistent with a research that established that a subscale of fatalism (pessimism) positively affects fear^13^. However, this is in disagreement with the studies carried out in the first months of the pandemic, as they report an inverse relationship between the fear of COVID-19 with fatalism in the fight against COVID-19^10^ and non-critical pessimistic fatalism^12^. It is pertinent to note that one of these studies used a scale not contextualized in COVID-19. This difference can be explained by the fear that motivates people to respond effectively to a threat or a certain stimulus, that is, a limit, a determination or a commitment. It represents a survival mechanism in everyday situations, that is, what protects us from certain facts characteristic of the human condition, which can become a potential danger^24^. However, extreme or persistent fear can cause negative psychological reactions, such as psychological stress^25^, anxiety and depression^26^. 

On the other hand, when considering that the average score of fear of COVID-19 obtained is higher than that reported in a study that used the same scale, in a Peruvian population^11^, but lower than that obtained among nurses who worked on the front line^25^, could be indicating that nursing professionals who work in direct care for people with COVID-19 are at greater risk of developing psycho-emotional problems.

Another predictor of fatalism reported in this study was concern about COVID-19. As no previous studies were found that evaluated this relationship specifically in this pandemic, this finding coincides with a survey carried out with Peruvian nurses that suggests that worry is a predictor of other psychological aspects such as anxiety^27^. In addition, another study was found that reported the correlation between worry and anxiety^14^. Likewise, it would be in agreement with a research carried out with people with cancer, in which it was found that the environmental concern of individuals reflects cognitive rationality and reasonableness in opposition to the negative association between fatalism and concern^28^.

The average score for concern about COVID-19 obtained in this research is similar to the score reported by a study carried out in a Peruvian population carried out in March 2020^14^, but slightly lower than that reported by another carried out with nurses, between April and July of the same year^27^. This shows that the result is consistent and confirms the findings of previous studies that used the same scale.

Finally, among the various variables analyzed in this investigation, the fact of having been diagnosed with COVID-19 was a predictor of fatalism in the two proposed models. Therefore, nurses who suffered from this disease had a higher fatalism score. As no studies were found that analyzed this association, it was not possible to compare this finding. However, when the results of the research were compared with studies carried out in the general population, having a history of cancer is a predictor of fatalism^29^.

The study reports that nurses have a moderate level of fatalism, a result that coincides with previous researches carried out in the general population^13^. This finding suggests the need for urgent implementation of measures to prevent and mitigate the psycho-emotional repercussions caused by the COVID-19 pandemic. Thus, everyday activities, contrary to a life project anchored in progress, appear tragic: they live between joy and pain, remembering that life is also a work of art, with all its ambiguity^24^. Thus, when everyday life hurts or is felt as anger, “only art has the power to transform what is horrible and absurd in existence and make life pleasant and possible”^30^. 

The findings of this study highlight the need for authorities, health and nursing establishments to consider fear, worry and fatalism as occupational diseases, in order to implement strategies to promote mental health and prevent psycho-emotional problems.

We suggest the implementation of educational and psycho-emotional interventions to reduce fatalism - aimed at nursing professionals diagnosed with COVID-19 who present high levels of fear or concern about this disease - through communication strategies that promote follow-up, due to the fact that of “being-together”, although it does not overcome death, at least it allows to relativize it and witness that life lasts^1^. Likewise, it is recommended to carry out research that assess the association of fatalism with other possible associated factors, such as spirituality, resilience and optimism, or its influence on the adoption of preventive and health promotion measures. The preparation of health teams in the development of emotional coping skills both in the face of acute and chronic stress is very important, avoiding an undesirable impact on the mental health of the professionals.

Thus, reassessing art allows rescuing the artist that exists in every human being, in the nurse, because nursing is a profession, science and art, “the most beautiful of the arts”^31^, in its confrontation in the face of the tragic and fruitful of so many life lessons, considering that the end of a world is not the end of the world, because we can build other possible worlds in the daily work through and after the pandemic, towards a dignified and healthy life.

The study had some limitations. As nurses from different regions of Peru were included, data collection was performed using a virtual form. Therefore, the information should be considered as self-reported. No previous researches were found that evaluated the factors associated with fatalism in nurses in the local and international context. Thus, the results were compared with studies performed in the general population. Finally, the results can be affected by selection bias due to the type of sample chosen, which must be considered when interpreting the findings. 

## Conclusion

In conclusion, the study presents evidence that concern, fear and the fact of having been diagnosed with COVID-19 can predict fatalism in nursing professionals through a model that explains a third of the total variability. Likewise, nurses had a moderate level of fatalism and a low level of fear and concern about COVID-19.

To reduce the fatalistic thinking of nursing professionals in the context of pandemics, it is suggested the implementation of interventions in the management of fear and concern that also strengthen individual and collective coping strategies. Thus, the fatal consequences of COVID-19 can be prevented and mental health promoted.

## References

[1] Maffesoli M (2020). Crise sanitária, crise civilizacional. https://www.cartapotiguar.com.br/2020/03/22/crise-sanitaria-crise-civilizacional.

[2] Organización Mundial de la Salud (2020). COVID-19 disrupting mental health services in most countries, WHO survey.

[3] Cabarkapa S, Nadjidai SE, Murgier J, Ng CH (2020). The psychological impact of COVID-19 and other viral epidemics on frontline healthcare workers and ways to address it: A rapid systematic review. Brain Behav Immun Health.

[4] De Kock JH, Latham HA, Leslie SJ, Grindle M, Munoz SA, Ellis L (2021). A rapid review of the impact of COVID-19 on the mental health of healthcare workers: implications for supporting psychological well-being. BMC Public Health.

[5] Ruta F, Dal Mas F, Biancuzzi H, Ferrara P, Della Monica A (2021). Covid-19 and front-line nurses' mental health: a literature review. Prof Inferm.

[6] Sher L (2020). The impact of the COVID-19 pandemic on suicide rates. QJM.

[7] Almaghrebi AH (2021). Risk factors for attempting suicide during the COVID-19 lockdown: Identification of the high-risk groups. J Taibah Univ Med Sci.

[8] Mejia CR, Rodríguez-Alarcón JF, Carbajal M, Pérez-Espinoza P, Porras-Carhuamaca LA, Sifuentes-Rosales J (2020). Fatalismo ante la posibilidad de contagio por el coronavirus: Generación y validación de un instrumento (F-COVID-19). Kasmera.

[9] Mejia CR, Quispe-Sancho A, Rodriguez-Alarcon JF, Ccasa-Valero L, Ponce-López VL, Varela-Villanueva ES (2020). Factors associated with fatalism in the face of COVID-19 in 20 Peruvian cities in March 2020. Rev Habanera Cienc Méd.

[10] Hayes J, Clerk L (2021). Fatalism in the Early Days of the COVID-19 Pandemic: Implications for Mitigation and Mental Health. Front Psychol.

[11] Huarcaya J, Villarreal D, Podestà A, Luna MA (2020). Psychometric Properties of a Spanish Version of the Fear of COVID-19 Scale in General Population of Lima, Peru. Int J Ment Health Addict.

[12] Bachem R, Tsur N, Levin Y, Abu-Raiya H, Maercker A (2020). Negative Affect, Fatalism, and Perceived Institutional Betrayal in Times of the Coronavirus Pandemic: A Cross-Cultural Investigation of Control Beliefs. Front Psychiatry.

[13] Özdil K, Büyüksoy GDB, Çatiker A (2021). Fatalism, fear, and compliance with preventive measures in COVID-19 pandemic: A structural equation modeling analysis. Public Health Nurs.

[14] Rodríguez TC, León JV, Palomino MB (2021). Design and validation of a scale to measure worry for contagion of the COVID-19 (PRE-COVID-19). Enferm Clínica.

[15] Jimenez T, Restar A, Helm PJ, Cross RI, Barath D, Arndt J (2020). Fatalism in the context of COVID-19: Perceiving coronavirus as a death sentence predicts reluctance to perform recommended preventive behaviors. SSM Popul Health.

[16] Wasserman D, Iosue M, Wuestefeld A, Carli V (2020). Adaptation of evidence-based suicide prevention strategies during and after the COVID-19 pandemic. World Psychiatry.

[17] Dunn TJ, Baguley T, Brunsden V (2014). From alpha to omega: A practical solution to the pervasive problem of internal consistency estimation. Br J Psychol,.

[18] Maher JM, Markey JC, Ebert-May D (2013). The Other Half of the Story: Effect Size Analysis in Quantitative Research. CBE Life Sci Educ.

[19] Kerlinger FN, Pedhazur EJ, Pedhazur EJ (1997). Multiple Regression in Behavioral Research.

[20] Moral RA, Hinde J, Demétrio C (2017). Half-Normal Plots and Overdispersed Models in R: The hnp Package. J Stat Software.

[21] Breusch TS, Pagan AR (1979). A Simple Test for Heteroscedasticity and Random Coefficient Variation. Econometrica.

[22] Fox J, Monette G (1992). Generalized Collinearity Diagnostics. J Am Stat Assoc.

[23] R Core Team (2021). R: The R Project for Statistical Computing.

[24] Maffesoli M (2020). Pensar o (im)pensável: Instituto Ciência e Fé e PUCPRESS debatem a pandemia com Michael Maffesoli.

[25] Labrague LJ, de Los Santos JAA (2021). Fear of COVID-19, psychological distress, work satisfaction and turnover intention among frontline nurses. J Nurs Manag.

[26] Yildirim M, Arslan G, Özaslan A (2020). Perceived Risk and Mental Health Problems among Healthcare Professionals during COVID-19 Pandemic: Exploring the Mediating Effects of Resilience and Coronavirus Fear. Int J Ment Health Addict.

[27] Hong SJ (2020). Linking environmental risks and cancer risks within the framework of genetic-behavioural causal beliefs, cancer fatalism, and macrosocial worry. Health Risk Soc.

[28] Carranza RF, Mamani O, Chaparro JET, Farfán R, Gonzales NC (2021). Concern about COVID-19 and workload as predictors of anxiety in Peruvian nurses. Rev Cuba Enferm.

[29] Amuta AO, Chen X, Mkuu R (2017). The Effect of Cancer Information Seeking on Perceptions of Cancer Risks, Fatalism, and Worry Among a U S. National Sample. Am J Health Educ.

[30] Nietzsche F (2006). Origem da Tragédia.

[31] Nightingale F (1990). Notas sobre enfermería: qué es y qué no es.

